# Photoluminescence of the Au_38_(SR)_26_ nanocluster comprises three radiative processes

**DOI:** 10.1038/s42004-023-00819-3

**Published:** 2023-02-02

**Authors:** Lianshun Luo, Zhongyu Liu, Xiangsha Du, Rongchao Jin

**Affiliations:** Department of Chemistry, Carnegie Mellon University, Pittsburgh, PA 15213 USA

**Keywords:** Chemical synthesis, Photochemistry, Optical materials, Synthesis and processing

## Abstract

Photoluminescence of ultrasmall, atomically precise gold nanoclusters constitutes an area of significant interest in recent years for both fundamental research and biological applications. However, the exploration of near-infrared photoluminescence of gold nanoclusters is still in its infancy due to the limitations of synthetic methods and characterization techniques. Herein, the photoluminescence properties of an Au_38_(PET)_26_ (PET = 2-phenylethanethiolate) nanocluster are investigated in detail. The Au_38_(PET)_26_ exhibits an emission peak at 865 nm, which is revealed to be a mix of fluorescence, thermally activated delayed fluorescence, and phosphorescence via the combined analyses of time-resolved and temperature-dependent photoluminescence measurements. The quantum yield of Au_38_(PET)_26_ is determined to be 1.8% at room temperature under ambient conditions, which increases to above 90% by suppressing the non-radiative relaxation pathway at a cryogenic temperature (80 K). Overall, the results of this work discover the coexistence of three radiative processes in thiolate-protected Au nanoclusters and will pave the way for understanding the intriguing photoluminescence properties of gold nanoclusters in future studies.

## Abstract (web summary)

Atomically precise gold nanoclusters display useful photoluminescence properties, but limitations in synthetic methods and characterization techniques have hindered their detailed exploration. Here, a Au_38_(PET)_26_ nanocluster is found to exhibit fluorescence, phosphorescence, and thermally activated delayed fluorescence emissions, with a significant enhancement in photoluminescence intensity at cryogenic temperatures owing to the suppression of nonradiative pathways.

## Introduction

Atomically precise gold nanoclusters (NCs) have attracted great attention owing to their well-defined compositions, structures and elegant properties^1–8^. Particularly, the photoluminescence (PL) properties of Au NCs have drawn increasing interest in recent years^9–15^. The atomic precision of Au NCs offers a great opportunity to eliminate the polydispersity-induced uncertainty and further map out the correlation between their structures and PL properties for those structurally characterized NCs^1^. Compared to the other common PL nanomaterials, Au NCs possess several unique merits, such as their ultrafine size (<2 nm), good biocompatibility, and excellent stability, which make this class of materials quite promising for PL-related applications including bioimaging, sensing, and cancer therapy^16–24^.

Many thiolate-protected Au NCs have been synthesized in recent years, and some of them have been shown to display intriguing PL characteristics. To improve the quantum yield (QY) of Au NCs, several strategies have been developed, such as heterometal doping^9^, crystallinity and ligand engineering^1,16^. Xie’s group reported aggregation-induced emission in the Au_22_(SR)_18_ NC, which gave rise to luminescence at ∼665 nm with a relatively high QY of ~8%^25^. Lee’s group further reported that the QY of Au_22_(SR)_18_ can be improved to above 60% by rigidifying the cluster’s shell with tetraoctylammonium cations^26^. This surface-regulated PL enhancement method was also investigated by Jin^27^, Millstone^28^, and Wu groups^29^. The Tsukuda group revealed a dramatic QY enhancement of Au_25_(SR)_18_ NCs by stiffening the icosahedral Au_13_ core via heterometal doping^30^, and this doping-induced QY enhancement strategy was also previously investigated by Bakr and Wu groups^31,32^. Li et al recently reported the bright emission of Au_38_S_2_(SR)_20_ in the near-infrared (NIR) region (900 nm) with QY up to 15% under ambient conditions^33^. Jin’s group recently reported dual emission of Au_42_(SR)_32_ in the NIR region with a QY of 11.9% and demonstrated the dipolar interaction-induced enhancement of intersystem crossing from singlet to triplet excited state^34^. Except for some special cases^25,26,33–36^, most of the Au NCs still have low PL QY (<1%), especially in the NIR region, which limits their biological applications. In addition, the PL characteristics of many Au NCs have not been fully investigated yet. Thus, much effort is still required in characterizing the PL and understanding the mechanisms in Au NCs.

Herein, we report the intriguing PL of a Au_38_(PET)_26_ (PET = 2-phenylethanethiolate) NC. Although this nanocluster was previously synthesized by Wu’s group^37^, there is no report yet on the detailed PL studies, which motivates our current work. The Au_38_(PET)_26_ NC shows an emission peak centered at 865 nm with a QY of 1.8% at room temperature. Detailed analyses indicate that fluorescence, phosphorescence, and thermally activated delayed fluorescence (TADF) emissions are present in Au_38_(PET)_26_. When the temperature decreases from 298 to 80 K, the vibrations of staple motifs are dampened, which suppresses the nonradiative pathway significantly, thus the PL intensity is enhancement by more than 50 times (i.e., approaching the near-unity QY). Meanwhile, both the fluorescence and TADF disappear in Au_38_(PET)_26_ as the temperature decreases, which is ascribed to the suppressed reverse intersystem crossing (RISC) from triplet (T_1_) to singlet (S_1_) excited state.

## Results and discussion

The Au_38_(PET)_26_ nanocluster was synthesized by a NHC-mediated synthetic method (NHC = N-heterocyclic carbene) reported by our group recently (see Supporting Information for details)^34^. The crude product was purified by thin-layer chromatography (Supplementary Fig. S1). On a note, previous work by Xia et al.^37^ obtained the Au_38_(PET)_26_ by reacting Au_25_(PET)_18_ with acetic acid, which was a transformation process, but our current method is a bottom-up synthesis.

**Fig. 1:**
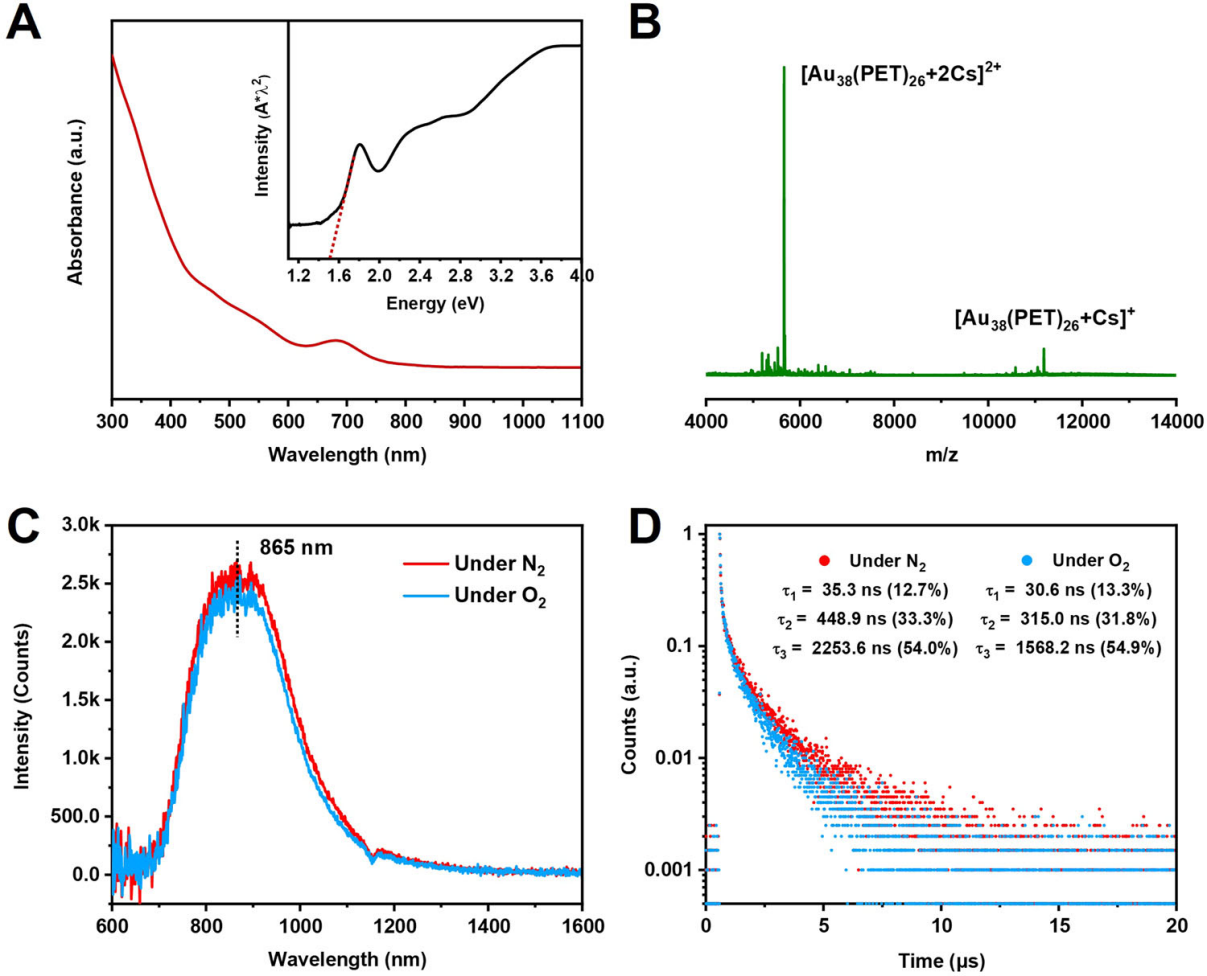
Mass spectrum and optical characterization of Au_38_(PET)_26_. **A** UV-Vis absorption spectrum of Au_38_(PET)_26_ in dichloromethane (DCM). The inset shows the absorption spectrum on the photon energy scale. **B** ESI mass spectrum of Au_38_(PET)_26_. **C** PL spectra of Au_38_(PET)_26_ in DCM under N_2_ and O_2_, respectively. **D** PL decay profiles of Au_38_(PET)_26_ in DCM under N_2_ and O_2_, respectively. For PL measurements: excitation at 400 nm (slit width 8 nm), and emission slit width 8 nm.

The UV-vis absorption spectrum of Au_38_(PET)_26_ (Fig. 1A) exhibits three peaks at 468, 560, and 680 nm, which are consistent with the previous report by Wu’s group^37^. The absorption edge of Au_38_(PET)_26_ is 1.5 eV (see the inset of Fig. 1A), which corresponds to the HOMO-LUMO gap and is consistent with the electrochemical gap^37^. To verify the formula, electrospray ionization (ESI) mass spectrometry was performed by adding cesium acetate to the cluster solution. The ESI mass spectrum (Fig. 1B) displays two prominent peaks at *m/z* 5658.9 and *m/z* 11185.0, which are assigned to [Au_38_(PET)_26_ + 2Cs]^2+^ (calculated *m/z*: 5659.3 by the formula, deviation: 0.4) and [Au_38_(PET)_26_ + Cs]^+^ (calculated *m/z*: 11185.6, deviation: 0.6), respectively. The close match between the experimental and calculated *m/z* values confirms the Au_38_(PET)_26_ formula. In addition, Au_38_(PET)_26_ is charge-neutral, evidenced by the observation that the charges in the adducts are equal to the Cs^+^ numbers (Fig. 1B). Meanwhile, Au_38_(PET)_26_ possesses high stability, as revealed by UV-vis absorption spectroscopic monitoring for 7 days (Supplementary Fig. S2) and no sign of degradation.

**Fig. 2:**
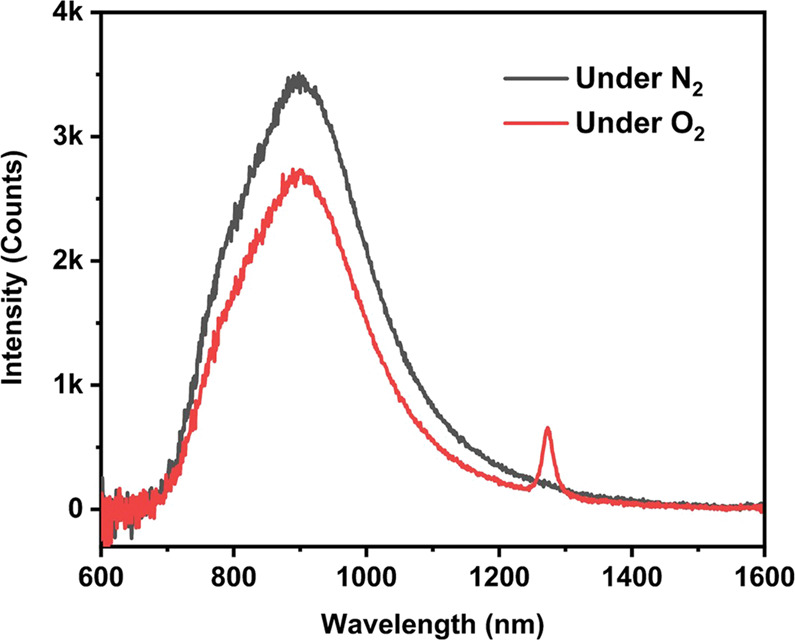
PL spectrum of Au_38_(PET)_26_ in deuterated chloroform under N_2_ and O_2_, respectively. The sharp peak at 1272 nm is from the emission of singlet oxygen (^1^O_2_, phosphorescence) due to sensitization of triplet ^3^O_2_ by the triplet state of Au_38_(PET)_26_ after photoexcitation.

The PL spectrum of Au_38_(PET)_26_ in dichloromethane (DCM) is shown in Fig. 1C, with the peak centered at 865 nm and a Stokes’ shift of 0.39 eV (i.e., 1241/680−1241/865 = 0.38 eV). The overall integrated intensity of PL was somewhat suppressed under pure O_2_ compared to N_2_, and the appearance of singlet oxygen (^1^O_2_) signal centered at 1272 nm (sharp phosphorescence emission) can be readily observed when deuterated chloroform (as opposed to DCM) was used as the solvent (Fig. 2), implying the existence of Au_38_(PET)_26_ triplet state and its sensitization of triplet oxygen (ground state of O_2_) to singlet oxygen (excited state). The QY of Au_38_(PET)_26_ in DCM under ambient conditions is measured to be 1.8% (using an integrating sphere). The PL excitation spectrum for the emission at 865 nm tracks the absorption profile (Supplementary Fig. S3), suggesting that the emission arises from the HOMO-LUMO transition. The PL dynamics was further investigated by the multi-channel scaling (MCS) single photon counting technique. As shown in Fig. 1D, three lifetime components are needed to fit the decay of the 865 nm emission. Under N_2_, the lifetimes include 35.3 ns (12.7%, τ_1_), 448.9 ns (33.3%, τ_2_), and 2.3 μs (54.0 %, τ_3_), respectively; note that the percentage in the parentheses indicates the relative intensity of each component. Among the three components, τ_1_ and τ_2_ can be assigned as fluorescence (both prompt and delayed fluorescence), and the long lifetime τ_3_ should be the triplet-state emission. In addition, τ_3_ decreases distinctly from 2.3 μs to 1.6 μs under pure O_2_, further validating its phosphorescence nature. The PL spectrum can be deconvoluted into two Voigt profiles (Supplementary Fig. S4), which are fluorescence (including τ_1_ for the prompt fluorescence and τ_2_ for the delayed fluorescence) and phosphorescence (τ_3_), respectively.

**Fig. 3:**
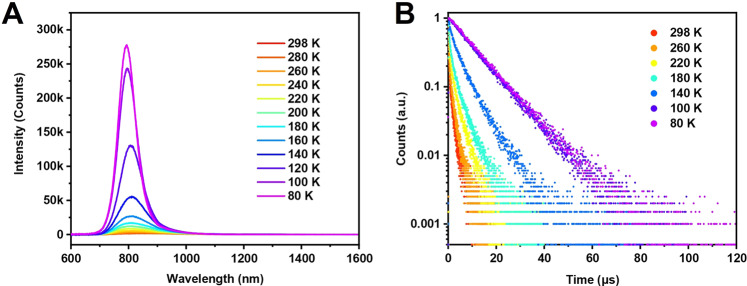
Cryogenic PL studies. **A** Temperature-dependent PL spectra of Au_38_(PET)_26_ in 2-MeTHF. **B** Decay profiles of Au_38_(PET)_26_ in 2-MeTHF at different temperatures. For PL measurements: excitation at 400 nm, slit width 5 nm, and emission slit width 5 nm.

The above results indicate quite complicated electron dynamics in Au_38_(PET)_26_. To gain further insight into the origin of PL, temperature-dependent PL spectra for Au_38_(PET)_26_ were measured from room temperature down to 80 K. The nanocluster was dissolved in 2-methyltetrahydrofuran (2-MeTHF) in order to have the formation of clear ‘glass’ at cryogenic temperatures for optical measurements; of note, the PL in 2-MeTHF and DCM are almost identical (Supplementary Fig. S5). As shown Fig. 3A, the PL peak becomes sharper and slightly blue-shifted as the temperature decreases. Such a trend was observed in previous cryogenic measurements on Au_25_(PET)_18_, Au_38_(PET)_24_, etc^27,38^. Meanwhile, the integrated intensity of the PL peak increases monotonically by ~50 times from room temperature to 80 K, which means that the PLQY is over 90% at 80 K. The observed near-unity PLQY at 80 K implies an almost complete suppression of non-radiative relaxation pathway, which is similar to the Au NCs with a mono-cuboctahedral kernel (e.g., Au_23_(SR)_16_^−^)^39^. We note that the 50-fold enhancement of PL is not due to the absorption increase at 80 K because the cryogenic absorption measurements (Supplementary Fig. S6) showed only a 28% increase in absorbance at 400 nm (the excitation wavelength for PL). The temperature-dependent PL excitation spectra (Supplementary Fig. S7) of Au_38_(SR)_26_ are essentially unchanged with decreasing temperature and all show similar spectral profiles as that of the room-temperature absorption spectrum (Fig. 1A). Therefore, the observed PL emission comes from the first excited state (singlet and triplet) over the temperature range.

Based on the above discussions, the 35 ns lifetime at room temperature should stem from the radiative relaxation of the first singlet excited state (S_1_), while the 2.3 μs should be from the radiative relaxation of the first triplet excited state (T_1_). To unravel the origin of the 448 ns component, time-resolved PL measurements were carried out in the 298−80 K temperature regime to understand the excited state electron dynamics. The obtained decay curves are plotted in Fig. 3B and Supplementary Fig. S8, and the fitting results are listed in Table 1. As the temperature decreases from room temperature down to 140 K, all of the three components become longer and the percentage of τ_3_ increases rapidly, while the percentages of τ_1_ and τ_2_ both decrease. Interestingly, when the temperature is lower than 140 K, only one component (i.e., τ_3_) is remained, whereas the other two radiative processes are suppressed at this cryogenic temperature; thus, we ascribe the observed τ_2_ to a TADF process. Generally speaking, TADF requires efficient intersystem crossing (S_1_ to T_1_) and a very small gap (<0.2 eV) between S_1_ and T_1_ so that thermal energy can repopulate the S_1_ state by a ‘uphill’ transfer of T_1_ population (i.e. RISC). The TADF in Au_38_(PET)_26_ implies the closely spaced S_1_ and T_1_ states and efficient populating in this nanocluster.

**Table 1:** Fitted lifetimes of time-resolved PL decays of Au_38_(PET)_26_ under different temperatures.

Temperature	τ_1_ (ns)	τ_2_ (ns)	τ_3_ (μs)
298 K	90.5 (15.5%)	692.3 (42.3%)	2.4 (42.2%)
260 K	94.1 (11.7%)	666.8 (29.2%)	2.2 (59.1%)
220 K	182.2 (10.3%)	923.6 (21.3%)	3.4 (68.4%)
180 K	273.8 (7.7%)	1473.2 (30.4%)	4.9 (61.9%)
140 K	775.6 (4.2 %)	2923.9 (25.4%)	7.0 (70.4%)
100 K	None	None	10.5 (100%)
80 K	None	None	10.9 (100%)

The percentages indicate the photon number% of the components.

**Fig. 4:**
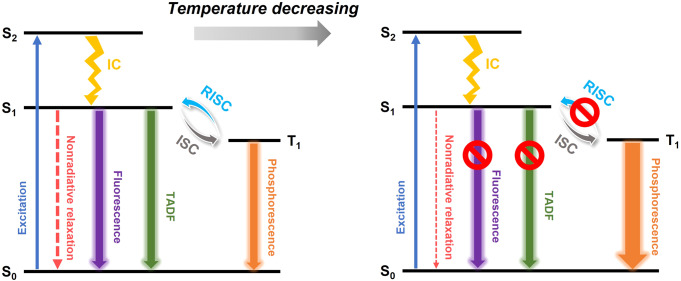
Proposed emission mechanism of Au_38_(PET)_26_. (IC internal conversion, S_0_ ground state, S_1_ and S_2_ are the excited states, T_1_ the lowest triplet state).

The proposed PL mechanism is shown in Fig. 4. At room temperature, three radiative processes, including the fluorescence, TADF and phosphorescence, contribute to the measured PL peak and result in the three components observed in the time-resolved PL measurements. As the temperature goes down, the overall PL intensity increases because of the suppression of nonradiative relaxation. When the temperature is lower than 140 K, the thermally activated reverse intersystem crossing (RISC) is suppressed and thus only phosphorescence from the T_1_ state remains.

Here, a question arises: why did the fluorescence and TADF disappear together at 140 K? We explain this from both the thermodynamic and kinetic aspects. On one hand, according to Hund’s rule, the T_1_ state is more thermodynamically stable than the S_1_ state, which makes T_1_ population more favored at low temperatures. On the other hand, the kinetics in Fig. 4 can be approximately described by Eq. (1) below (note: the nonradiative process is omitted)^40^. In previous studies of group 11 metal complexes, the ISC rate constant *k*_*ISC*_ was found to be much larger than the S_1_ radiative rate constant *k*_*S1*_^41,42^_._ In our case, Au_38_(PET)_26_ has much more metal atoms than a metal complex, and the stronger spin-orbit coupling could lead to a larger *k*_*ISC*_^11^. With the assumption of *k*_*ISC*_ >> *k*_*S1*._, at 140 K, if we make an approximation that the nonradiative relaxation can be neglected, the emission rate can be written as Eq. (2). Apparently, if the *k*_*RISC*_ becomes suppressed at low temperatures (i.e., *k*_*RISC*_ goes to near zero), the emission will be almost from the T_1_ state only. Of note, the approximation of neglecting nonradiative process is reasonable because the PLQY is between 40% and 100 % when the temperature is below 140 K, suggesting that the radiative rate constant *k*_*r*_ and nonradiative rate constant *k*_*nr*_ values are at the same order of magnitude.

1 $${S}_{0}+hv\mathop{\longleftarrow }\limits^{{k}_{T1}}{T}_{1}{\mathop{\rightleftarrows}\limits_{{k}_{ISC}}^{{{k}_{RISC}}}}{S}_{1}\mathop{\longrightarrow }\limits^{{k}_{S1}}{S}_{0}+hv$$

2 $$\frac{{{{{{\boldsymbol{d}}}}}}({{{{{\boldsymbol{hv}}}}}})}{{{{{{\boldsymbol{dt}}}}}}}=\left(\frac{{{{{{{\boldsymbol{k}}}}}}}_{{{{{{\boldsymbol{RISC}}}}}}}\,{{{{{{\boldsymbol{k}}}}}}}_{{{{{{\boldsymbol{s}}}}}}1}}{{{{{{{\boldsymbol{k}}}}}}}_{{{{{{\boldsymbol{ISC}}}}}}}+{{{{{{\boldsymbol{k}}}}}}}_{{{{{{\boldsymbol{s}}}}}}1}}+{{{{{{\boldsymbol{k}}}}}}}_{{{{{{\boldsymbol{T}}}}}}1}\right)\left[{{{{{{\boldsymbol{T}}}}}}}_{1}\right]$$

## Conclusion

In summary, this work reports the synthesis of Au_38_(PET)_26_ via an NHC-mediated strategy and the probing of its PL properties. The Au_38_(PET)_26_ exhibits an emission peak at 865 nm and the PLQY is 1.8% at room temperature under ambient conditions. Temperature-dependent PL measurements reveal three components in the observed emission, namely fluorescence, TADF, and phosphorescence. Additionally, temperature-dependent PL measurements show an almost complete suppression of non-radiative relaxation pathway, improving the PLQY to above 90% at 80 K.

## Methods

### Synthesis of chloro(dimethylsulfide)gold(I) (AuCl(SMe_2_))

HAuCl_4_·3H_2_O (500 mg, 1.27 mmol) was dissolved in ethanol (20 mL), followed by the addition of SMe_2_ (280 μL, 3.81 mmol) and vigorous stirring for 2 h. After stirring, the white precipitate was collected by centrifugation, which was washed with ethyl ether and finally dried to give the product as a white powder.

### Synthesis of 1,3-diisopropylbenzimidazolium bromide (iPr2-bimy·HBr)

Benzimidazole (1.18 g, 10 mmol) and K_2_CO_3_ (760 mg, 5.5 mmol) were added into acetonitrile (8 mL) and rapidly stirred at ambient temperature for 1 h. Following that, 2-bromopropane (5.4 mL, 57.5 mmol) was added to the suspension, and the reaction mixture was vigorously stirred under reflux conditions for 24 h, followed by the addition of the second portion of 2-bromopropane (5.4 mL, 57.5 mmol). The reaction mixture was vigorously stirred under reflux for additional 48 h. After removing the solvent under reduced pressure, DCM was added to the residue, and the upper supernatant after centrifugation was collected. The solvent of the supernatant was removed under reduced pressure to produce a spongy solid, which was washed by ethyl acetate to afford the desired product as a white powder.

### Synthesis of NHC-Au-Br complex (iPr2-bimy·AuBr)

^i^Pr_2_-bimy·HBr (1337.4 mg, 4.725 mmol), AuCl(SMe_2_) (1393.4 mg, 4.725 mmol), and K_2_CO_3_ (653.5 mg, 4.725 mmol) were added into acetone (20 mL) and vigorously stirred under reflux conditions for 2 h. After stirring for 2 h, the solvent in the suspension was removed under reduced pressure. DCM was added to the residue, and the upper supernatant after centrifugation was collected. The solvent of the supernatant was removed under reduced pressure to give the solid product, which was washed with pentane and finally dried to afford the desired product as a gray powder.

### Synthesis of Au_38_(PET)_26_

^i^Pr_2_-bimy·AuBr (120 mg, 0.25 mmol) and PET (67 μL, 0.5 mmol) were dissolved in a mixture of chloroform (15 mL) and ethanol (5 mL), and the mixture gradually turned cloudy white within 10 min of stirring (Au^I^-PET was formed). Following that, the suspension was reduced to NCs with the addition of (CH_3_)_3_CNH_2_·BH_3_ (434 mg, 5 mmol), indicated by the formation of a brown solution. The reaction was continued for 72 h, and then the solvent was removed under reduced pressure. The mixture of Au NCs was thoroughly washed with methanol, extracted with DCM, and concentrated for TLC separation. The mixture of Au NCs was pipetted on the TLC plate, and the separation was conducted in a developing tank (developing solvent 1:1 (v/v) DCM:*n*-hexane) (Supplementary Fig. S1). The red-brown band corresponding to Au_38_(PET)_26_ was cut off and dissolved in DCM for characterization.

### Steady-state UV-Vis-NIR measurements

UV-Vis-NIR spectra of gold nanoclusters were collected on a UV-3600 Plus UV-VIS-NIR spectrophotometer (Shimadzu).

### Steady-state photoluminescence and cryogenic measurements

Steady state photoluminescence spectra were measured on a FLS-1000 spectrofluorometer (Edinburgh). Visible PL was measured using a photomultiplier tube (PMT) as the detector (up to ~850 nm). Near-infrared PL was measured using a wide-range InGaAs detector (600–1700 nm) cooled to −80 °C by liquid nitrogen. The low temperature system was home-built, which included the FLS-1000 spectrofluorometer, a vacuum pump, an Optistat CF2 cryostat (Oxford Instruments), and a temperature controller. Liquid helium was used as the cryogen. The QY of Au_38_(PET)_26_ in DCM was measured by an integrating sphere.

## Supplementary information

Supplementary Information

## Data Availability

All relevant data are available from the authors by request.
