# Supplementary material for: Photoluminescence of the Au38(SR)26 nanocluster comprises three radiative processes
Source: Commun Chem. 2023 Feb 2;6:22. doi: 10.1038/s42004-023-00819-3 (PMC9894927; doi:10.1038/s42004-023-00819-3)
Supplement: Supplementary file 1 — Supplementary Information [file 42004_2023_819_MOESM1_ESM.pdf]

Supplementary Material

Photoluminescence of the $Au_{38}(SR)_{26}$ Nanocluster Comprises Three Radiative Processes

Lianshun Luo[$, 1], Zhongyu Liu[$, 1], Xiangsha Du[1], and Rongchao Jin[*, 1]
[1]Department of Chemistry, Carnegie Mellon University, Pittsburgh PA 15213, United States

*To whom correspondence should be addressed: rongchao@andrew.cmu.edu
[$] L.L. and Z.L. contributed equally to this work.

Chemicals

Tetrachloroauric (III) acid ($HAuCl_4 \cdot 3H_2O$, 99.999% metal basis, Aldrich), phenylethyl mercaptan (PET, >99%, Aldrich), borane-tert-butylamine complex ($(CH_3)_3CNH_2 \cdot BH_3$, 97.0%, Aldrich), Benzimidazole (98%, Aldrich), 2-bromopropane (99%, Aldrich), dimethyl sulfide (SMe_2, >99%, Aldrich), potassium carbonate (K_2CO_3, 99%, Aldrich), methanol, ethanol, ethyl ether, ethyl acetate, pentane, dichloromethane (DCM), chloroform, acetone, hexane and acetonitrile (HPLC grade for all solvents) were used as received. Thin-layer chromatography (TLC) plates were from iChromatography (silica gel, 250 μm).

Supporting Figures:

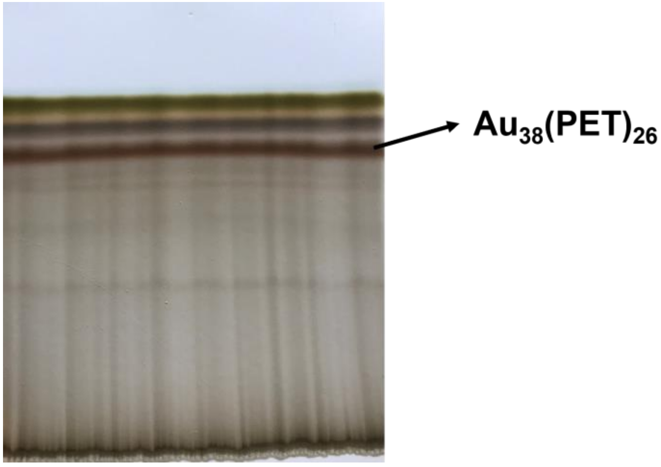

Figure S1. Thin Layer Chromatography (TLC) separation of $Au_{38}(PET)_{26}$ from the product mixture.

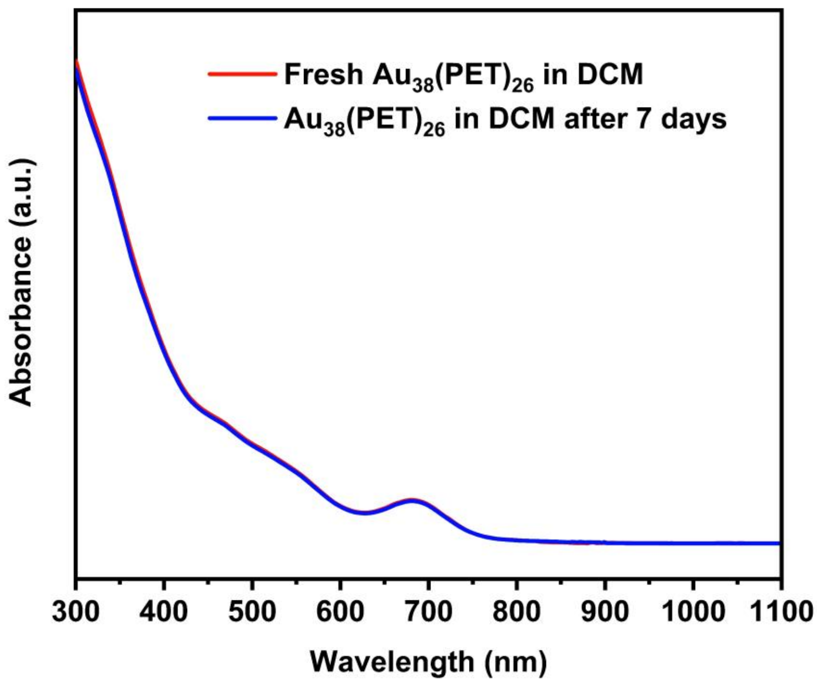

Figure S2. Stability evaluation of $Au_{38}(PET)_{26}$ in DCM by UV-vis absorption spectral comparison between 0 and 7 days (the two spectra are completely superimposable).

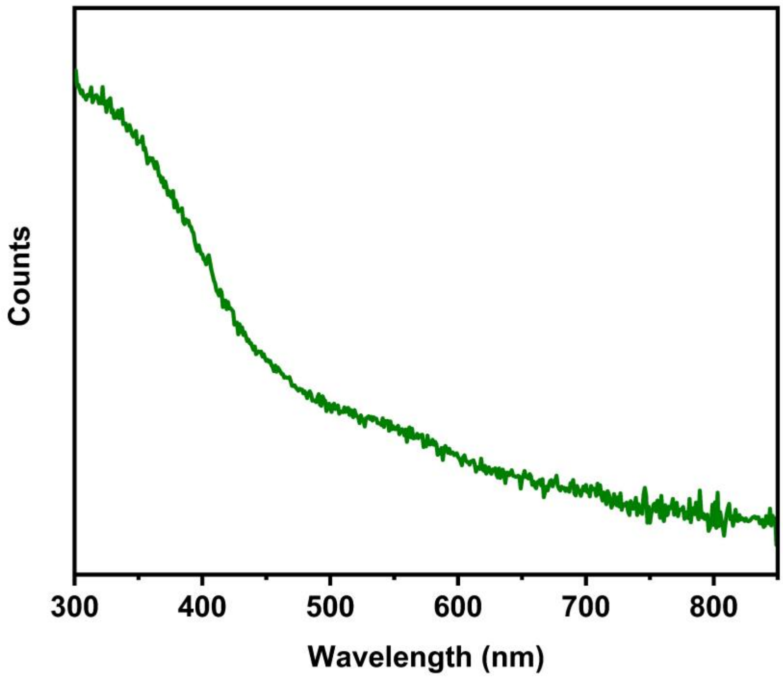

Figure S3. PL excitation spectrum for $Au_{38}(PET)_{26}$ in DCM at room temperature, measured at 865 nm PL. For PL measurements: slit width 8 nm, and emission slit 8 nm.

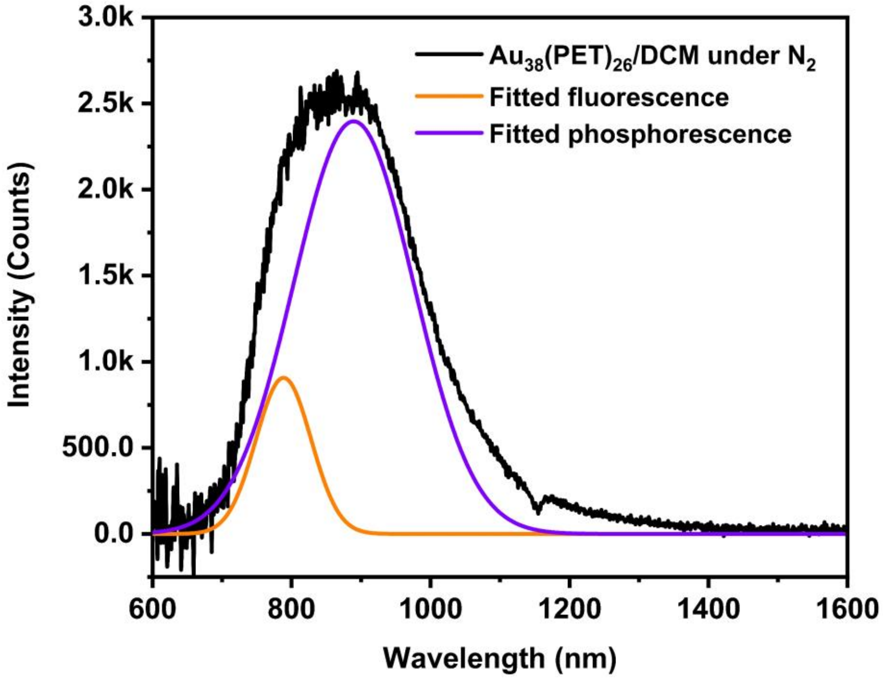

Figure S4. Deconvolution of the PL spectrum of $Au_{38}(PET)_{26}$ in DCM (black curve) into the fluorescence (orange curve) and phosphorescence (purple curve).

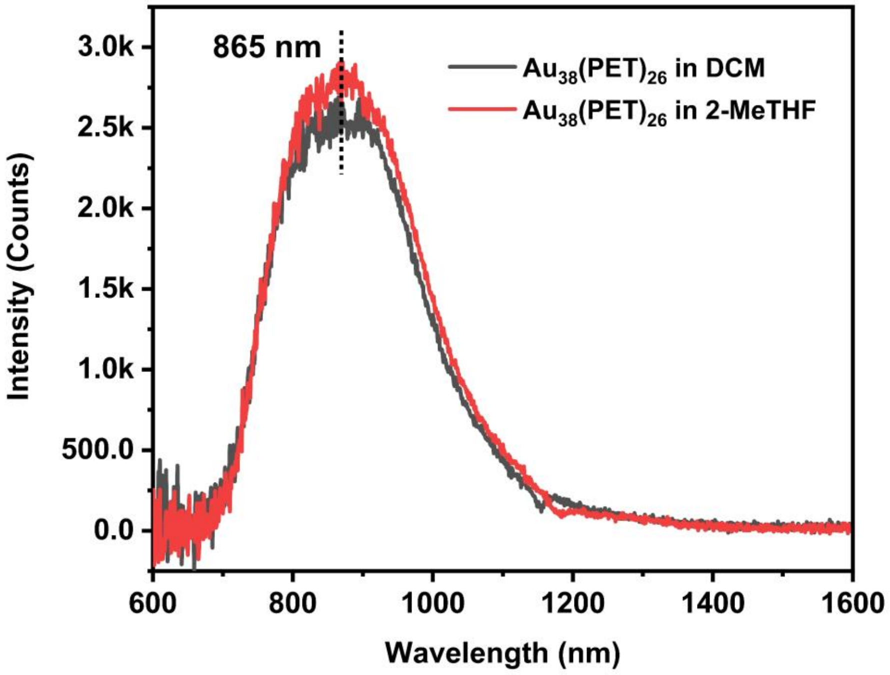

Figure S5. Comparison of PL spectra of $Au_{38}(PET)_{26}$ in DCM and 2-MeTHF. For PL measurements: excitation at 400 nm, slit width 8 nm, and emission slit 8 nm.

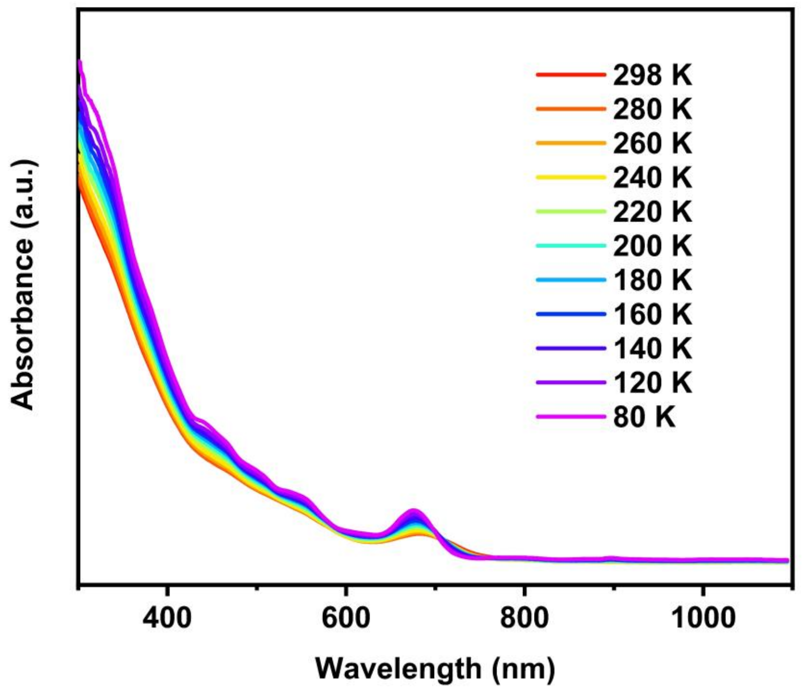

Figure S6. Temperature-dependent UV-vis absorption spectra of $Au_{38}(PET)_{26}$ in 2-MeTHF (solvent for clear 'glass' formation at cryogenic temperatures).

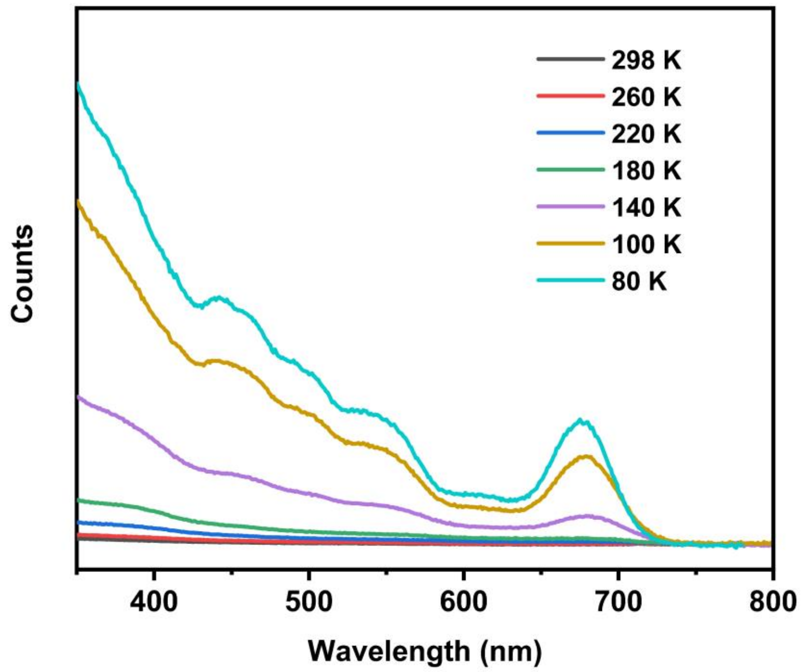

Figure S7. PL excitation spectra for $Au_{38}(PET)_{26}$ in 2-MeTHF at different temperatures. For PL measurements: slit width 5 nm, and emission slit 5 nm.

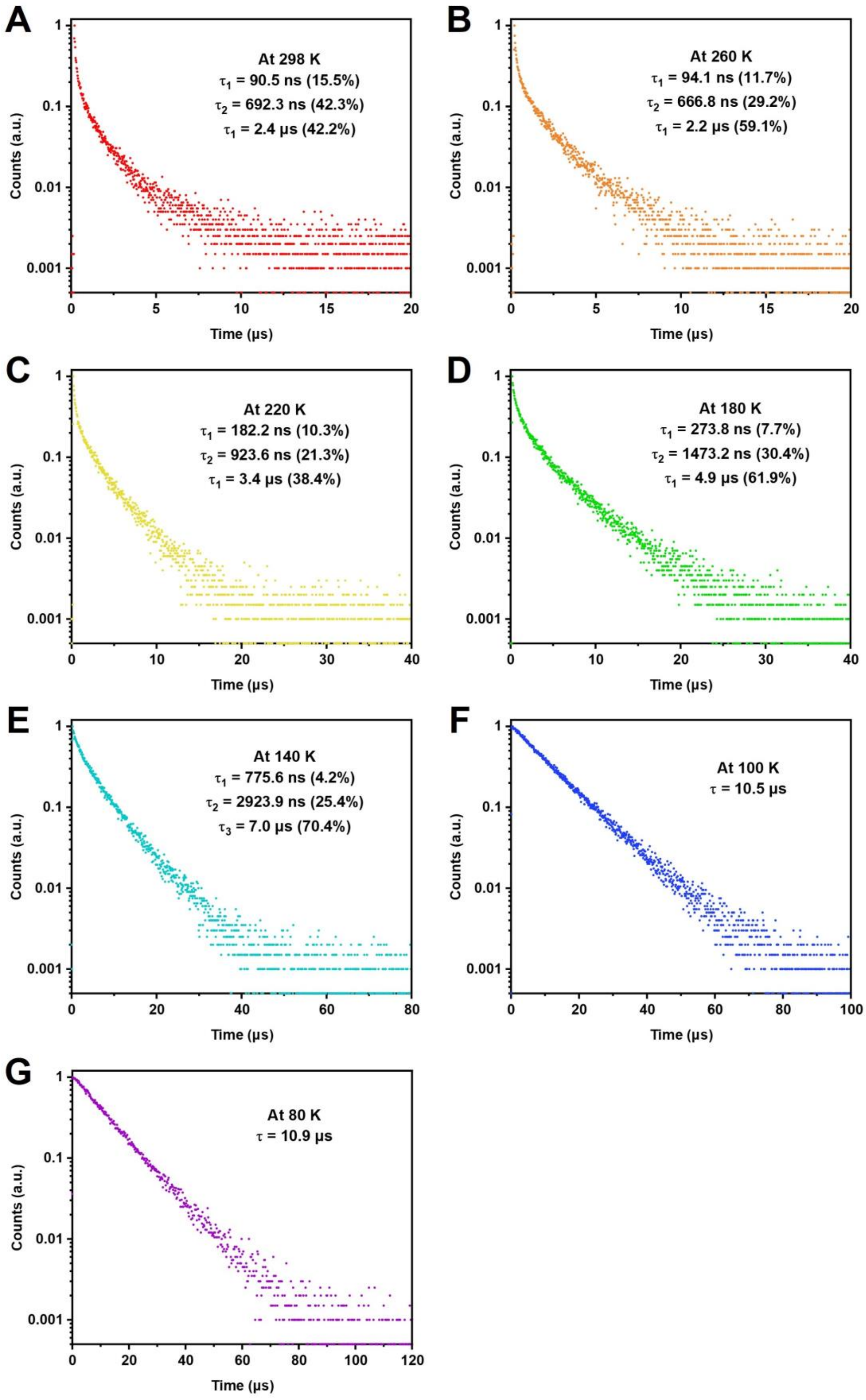

Figure S8. Decay profile of $Au_{38}(PET)_{26}$ in 2-MeTHF at (A) 298 K, (B) 260 K, (C) 220 K, (D) 180 K, (E) 140 K, (F) 100 K, and (G) 80 K. Insets: the fitted lifetimes and their amplitudes.
